# Guillain-Barré Syndrome, Greater Paris Area

**DOI:** 10.3201/eid1206.051369

**Published:** 2006-06

**Authors:** Valérie Sivadon-Tardy, David Orlikowski, Flore Rozenberg, Christiane Caudie, Tarek Sharshar, Pierre Lebon, Djillali Annane, Jean-Claude Raphaël, Raphaël Porcher, Jean-Louis Gaillard

**Affiliations:** * Hôpital Raymond Poincaré, Garches, France;; † Hôpital Saint-Vincent-de-Paul, Paris, France;; ‡ Hôpital Neurologique et Neurochirurgical Pierre Wertheimer, Lyon, France;; § Hôpital Saint-Louis, Paris, France

**Keywords:** Guillain-Barré syndrome, Campylobacter jejuni, cytomegalovirus, France, epidemiology, microbiology, dispatch

## Abstract

We studied 263 cases of Guillain-Barré syndrome from 1996 to 2001, 40% of which were associated with a known causative agent, mainly *Campylobacter jejuni* (22%) or cytomegalovirus (15%). The cases with no known agent (60%) peaked in winter, and half were preceded by respiratory infection, influenzalike syndrome, or gastrointestinal illness.

Guillain-Barré syndrome (GBS) is a state of acute flaccid paralysis thought, in most cases, to result from an aberrant immune response triggered by microbial infections (*1*). Studies in Western countries have reported evidence of recent infection with *Campylobacter jejuni* in 15% to 40% of GBS cases and with cytomegalovirus (CMV) in 5% to 20% of cases (*1**–**5*). Recent infection with Epstein-Barr virus (EBV) or *Mycoplasma pneumoniae* was less frequent (1%–2% each) (*1**–**5*). No agent was identified in 60% to 70% of cases, although the patients often had a history of respiratory or gastrointestinal infection (*1**,**2*).

Previous studies have failed to identify any clear seasonal distribution of GBS cases in Europe and North America. It has been suggested that this failure to demonstrate seasonality in GBS is because most prevalent antecedent infections have inverse seasonal distributions (*6*). We tested this hypothesis to provide new insight into infectious agents associated with GBS in Western countries.

## The Study

All GBS patients admitted to the medical intensive care unit of Raymond Poincaré Hospital in Garches, France, from January 1996 to December 2001 were included in the study. GBS was diagnosed according to the criteria of Asbury and Cornblath (*7*). The following data were collected at the time of hospital admission: time from onset of neurologic signs of GBS to admission; history of infections in the 2 months preceding the onset of neurologic signs; and time from the infectious event to onset of neurologic signs.

Serum samples were collected at hospital admission. Serum antibodies against *C*. *jejuni* and *M*. *pneumoniae* were assayed with complement fixation tests (Institut Virion, Würzburg, Germany); cutoff titers (*C*. *jejuni* 20; *M*. *pneumoniae* 80) were selected to give >95% specificity on the basis of data provided by the manufacturer. Serum samples were tested for immunoglobulin M (IgM) and IgG antibodies to CMV with the miniVIDAS system (bioMérieux, Marcy l'Etoile, France). IgG avidity was measured in samples positive for IgM by using the Enzygnost anti-CMV/IgG test (Dade Behring S.A., Paris la Défense, France) and 8 mol/L urea. Recent CMV infection was identified by detection of IgM with IgG avidity <35% (*8*). Serum antibodies against EBV were detected with commercial dot blot tests (ImmunoDOT EBV MONO M and G kits, GenBio, San Diego, CA, USA). Recent EBV infection was identified by detecting IgM antibodies to viral capsid antigen. IgM and IgG antibodies against gangliosides GM1 and GM2 were identified by an enzyme immunoassay (GanglioCombi, Bühlmann Laboratories AG, Schönenbuch, Switzerland) and an immunodot blot assay (*9*).

Statistical analyses were performed with the R 2.0.1 statistical package (R Development Core Team, Vienna, Austria). Groups were compared in pairs, and the Hochberg method for multiple testing was used to correct p values (*10*). Categorical variables were compared by Fisher exact test, and continuous variables were compared by Student *t* test or Wilcoxon rank sum test. Seasonal trends for GBS cases were analyzed by using the method of Jones et al. (*11*). The number of harmonics (seasonality periods) was determined by using the Akaike information criterion. All tests were 2-tailed, and a p value <0.05 was considered significant.

During the study period, 279 consecutive patients with GBS were admitted to our center. Sixteen patients were excluded because of missing clinical data or serum samples; 263 were included in the study. On the basis of an annual incidence of 1.2 to 1.9 GBS cases per 100,000 persons (*12*) in a population of 10.952 million people (*13*), we estimated that 130–210 GBS cases occurred annually in the greater Paris area during the study period. Thus, this study included 20%–30% of all estimated GBS cases in this area.

**Figure 1:**
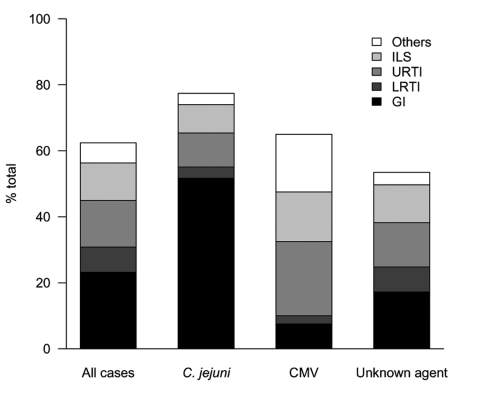
Distribution of preceding infectious events in patients with Guillain-Barré syndrome. ILS, influenzalike syndrome; URTI, upper respiratory tract infection; LRTI, lower respiratory tract infection; GI, gastrointestinal illness; CMV, cytomegalovirus infection.

We observed serologic evidence of recent infection with *C*. *jejuni* in 58 patients (21.9%), CMV in 40 (15.1%) patients, *M*. *pneumoniae* in 6 (2.3%) patients, and EBV in 3 (1.15%) patients. Recent infection with *C*. *jejuni* and CMV was observed in 1 patient. Thus, 106 cases (40%) had >1 known agent of GBS (known agent group), and 157 cases (60%) had no known agent (unknown agent group) (Table). Most patients in the *C*. *jejuni* group were male, were >50 years of age, had a history of gastrointestinal illness (Figure 1), and exhibited a severe motor form of GBS with serum IgG antibodies against ganglioside GM1. Patients in the CMV group were significantly younger (p<0.0001), more likely to have respiratory or influenzalike symptoms than gastrointestinal symptoms (p<0.0001) before the onset of GBS symptoms (Figure 1), and showed a longer time from first neurologic signs to hospital admission (p = 0.048). These patients rarely showed a pure motor form of GBS (p = 0.037) and frequently had IgM antibodies against GM2 but did not have IgG antibodies against GM1 (p<0.0001).

**Table:** Characteristics of patients with Guillain-Barré syndrome*

Characteristic	All patients	*Campylobacter jejuni*	CMV	Unknown agent	p value†	p value‡
No.	263	58	40	157		
Sex, no. (%)					0.53	0.31
Female	112 (43)	21 (36)	22 (55)	66 (42)		
Male	151 (57)	37 (64)	18 (45)	91 (58)		
Mean age, y (SD)	48.7 (18.3)	51.3 (19.1)	35.9 (12.0)	51.2 (17.9)	0.94	<0.0001
Mean days from infectious event to neurologic signs (IQR)	8 (4–15)	7 (4–14.2)	10 (4.5–18)	8 (5–15)	0.45	0.55
Mean days from neurologic signs to hospital admission (IQR)	5.5 (3–9)	4 (2.5–7	7 (3–11)	6 (3–10)	0.11	0.27
Infectious event, no. (%)					0.0048	0.25
None	99 (38)	13 (22)	14 (35)	73 (47)		
>1	164 (62)	45 (78)	26 (65)	84 (53)		
Infectious event, no. (%)					0.0049	0.026
GI	61 (37)	30 (67)	3(11)	27 (31)		
URTI	37 (23)	6 (13)	9 (35)	21 (25)		
LRTI	29 (12)	2 (4)	1 (4)	12 (14)		
Influenzalike	30 (18)	5 (11)	6 (23)	18 (21)		
Others	16 (10)	2 (4)	7 (27)	6 (7)		
Only motor symptoms, no. (%)	101 (39)	31 (54)	11 (28)	57 (37)	0.056	0.35
Mechanical ventilation, no. (%)	87 (33)	23 (40)	15 (38)	43 (27)	0.20	0.49
Antibodies to gangliosides, no. (%)						
GM1	30 (13)	24 (44)	0	5 (4)	<0.0001	0.59
GM2	15 (6)	0	15 (47)	0	1.0	<0.0001

*CMV, cytomegalovirus; SD, standard deviation, IQR, interquartile range; GI, gastrointestinal illness; URTI, upper respiratory tract infection; LRTI, lower respiratory tract infection. †Adjusted for unknown agent versus *C. jejuni*. ‡Adjusted for unknown agent versus CMV.

Patients in the unknown agent group were older than those in the CMV group (p<0.0001), less likely to have had a history of infectious events than patients in the *C*. *jejuni* group (p = 0.0048), and had a significantly different antiganglioside response than those in *C*. *jejuni* and CMV groups (p<0.0001 in each case) (Table). The unknown agent group had a higher proportion of patients with gastrointestinal illness than did the CMV group (p = 0.045) and a higher proportion of patients with respiratory tract or influenzalike symptoms than the *C*. *jejuni* group (p = 0.0024) (Figure 1).

No seasonal variation was found for all patients combined (data not shown). However, this apparent absence of variation masked a substantial seasonal difference for the known agent and unknown agent groups. In the known agent group, 60% of cases occurred in spring and summer; only 16% occurred in winter. In the unknown agent group, only 17% of cases occurred in summer; 37% occurred in winter.

**Figure 2:**
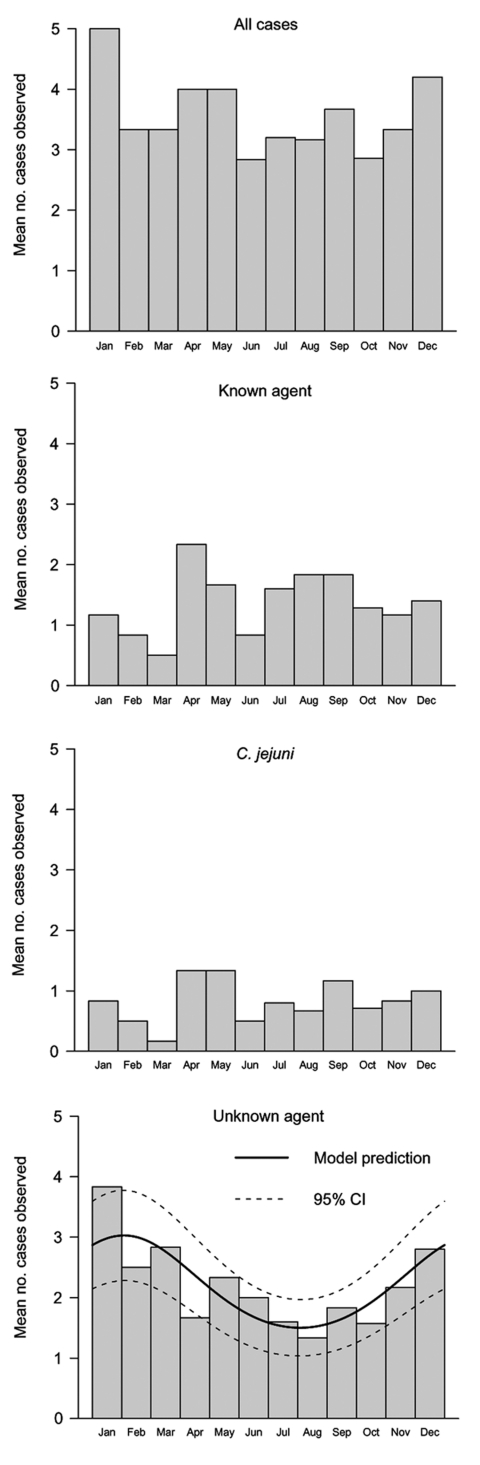
Seasonal distribution of preceding infectious agents by month for the study period (1996–2001). For the unknown agent group, the solid line represents the seasonal model prediction and the dashed lines represent its pointwise 95% confidence interval (CI).

We used the method of Jones et al. (*11*) to test the seasonality of incidence. No seasonality was detected for the groups all cases, known agent, and *C*. *jejuni* (Figure 2). For the unknown agent group, a model with 1 harmonic (annual seasonality) gave a significantly better fit than a model without harmonics (p = 0.0089, by likelihood ratio test); additional harmonics did not improve the fit of the model. Since no significant linear trend was found (p = 0.49), this element was removed for model prediction. This best-fit, single-harmonic model indicated that incidence was highest at the beginning of February and lowest at the beginning of August (Figure 2).

## Conclusions

This study provides new data about GBS patients not associated with known etiologic agents, which account for most patients in Western Europe (*2**,**14*). We have shown that GBS cases of unknown cause were more common in winter, with a peak incidence at the beginning of February. Moreover, in ≈50% of the patients, GBS symptoms were preceded by respiratory infection, influenzalike syndrome, or gastrointestinal illness. Together with the seasonality of cases, this finding suggests the involvement of winter infectious agents, probably respiratory or enteric viruses.
